# Anxiety symptoms during COVID-19 in older adults with psychiatric disorders

**DOI:** 10.3389/fpsyt.2022.1082807

**Published:** 2023-01-16

**Authors:** Elodie Pongan, Isabelle Rouch, Mathieu Herrmann, Catherine Perrot, Cécile Lebrun-Givois, Laurie Spirli, Chloé Briollet, Hélène Saint Martin, Bernard Laurent, Romain Bachelet, Hanane El Haouari, Aurélie Buisson, Arlette Edjolo, Jean-Michel Dorey

**Affiliations:** ^1^Memory Clinical and Research Center of Saint Etienne, Neurology Unit, University Hospital of Saint-Étienne, Saint-Étienne, France; ^2^Clinical and Research Memory Centre of Lyon, Hospices Civils de Lyon, Hôpital des Charpennes, Villeurbanne, France; ^3^INSERM, U1219, Bordeaux Population Health Center, University of Bordeaux, Bordeaux, France; ^4^Department of Aging Psychiatry, Hospital Le Vinatier, Bron, France; ^5^Memory Clinical and Research Center of Saint-Étienne, Geriatrics Unit, University Hospital of Saint-Étienne, Saint-Étienne, France; ^6^INSERM, U1028, CNRS, UMR 5292, Neuropain Team, Lyon Neuroscience Research Center, Lyon, France

**Keywords:** older patients, COVID-19, pandemic, mental health, anxiety, personality, coping strategies

## Abstract

**Objective:**

During the COVID-19 pandemic, older people and patients with psychiatric disorders had an increased risk of being isolated. The French National Authority for Health has recommended a reinforced follow-up of these patients. Cross-sectional studies reported an increased risk of developing anxiety and depression during pandemic. The aim of our study was to identify factors associated with higher anxiety during the pandemic in older patients with psychiatric disorders.

**Methods:**

STERACOVID is a multicenter cohort study with 117 patients followed-up by phone in two French geriatric psychiatry units. In this work, we used cross-sectional data from a prospective follow-up conducted between January and May 2021.

**Results:**

We found that coping strategies, personality, and living conditions were associated with general anxiety (GA) level during the pandemic period. Higher GA was associated with less positive thinking coping strategy, more avoidance strategies, a lower level of extraversion, a higher level of neuroticism, more time spent watching the news, a higher feeling of loneliness, and a lack of physical contact.

**Findings:**

Our study identified factors associated with a poorer experience of pandemic crisis. Special attention should be paid to patients with a high level of neuroticism and a high feeling of loneliness. Support could aim to help patients use more functional strategies: reducing avoidance strategies and increasing positive thinking. Finally, reducing time watching news could also be an interesting prevention perspective.

**Clinical trial registration:**

clinicaltrials.gov, identifier NCT04760795.

## 1. Introduction

In March 2020, an unprecedented pandemic has forced many countries to take drastic measures. As a result, policies restricting human contact and movement have been implemented worldwide. Previous epidemics and cross-sectional studies during the first wave of COVID-19 have shown that quarantines may have an impact on the mental health of populations, which can be as deleterious as the disease itself (1, 2).

Under this context, vulnerable populations such as older adults and people with psychiatric disorders, may require more attention and care. According to information from the World Health Organization (WHO), the COVID-19 affects the older population with a significantly high mortality rate. Because older adults are unfamiliar with new technologies and the use of video communications, social isolation resulting from COVID-19 restrictions were particularly marked for them. A literature review by Roy et al. (3) showed that isolation and loneliness were associated with significantly increased morbidity and mortality in the geriatric population. The review further identified several factors to alleviate emotional overwhelm including virtual interactions, physical activity, and watching the news in moderation. Another reason the lockdowns complicate the daily lives of older people is that many are dependent on their children or outside help (4). Furthermore, socio-economic conditions have been identified as a risk factor for psychological disorders following quarantine measures during previous epidemics (5), and the older adults are oftentimes in a financially precarious situation.

As seen with the older adults, patients with psychiatric disorders also have a higher risk of social isolation and financial vulnerability as well as medical comorbidities (6). A meta-analysis has shown that pre-existing mental disorders, particularly psychotic disorders, and mood disorders, as well as exposure to antipsychotic and anxiolytic treatments are associated with increased mortality from COVID-19 (7). Additionally, a cross-sectional study showed that pre-existing psychiatric condition was identified as a notable risk factor for poorer mental health during the COVID-19 crisis. A worsening of pre-existing psychiatric conditions and past exposure to trauma more precisely predicted increased suicidal ideation (8).

A large longitudinal study of three Dutch cohorts of adults (mean age 56 years) assessed the impact of COVID-19 on the mental health of individuals with and without depressive symptoms, anxiety disorder, and obsessive-compulsive disorder. Severity and chronicity of anxiety, depression, and obsessive-compulsive disorder were associated with poorer perceived mental health, greater fear of COVID-19, and reduced positive coping during the pandemic. Nevertheless, individuals with prior mental disorders did not show an increase in symptoms during the pandemic; conversely, individuals without prior mental disorders showed an increase in anxiety, depression, anxiety, and loneliness (9).

Few studies have been conducted on the mental health symptoms of older adults with psychiatric disorders during the pandemic. To our knowledge, there are no studies conducted after the first lockdown. A previous study suggested that elderly bipolar patients might have fewer psychiatric symptoms during COVID-19 than before the pandemic, however, several factors such as not having children, having a greater feeling of loneliness, lower control, passive coping style, and a high level of neuroticism were, in fact, associated with an increase in psychiatric symptoms during COVID-19 (10).

The aim of our study was to identify factors associated with higher anxiety during the COVID-19 pandemic in older patients with psychiatric disorders. We expect that:

(a) People who are more isolated, have less physical or digital contact, and spend more time watching the news should demonstrate greater anxiety during the COVID-19 crisis.
(b) Increased use of more active strategies, such as problem solving or seeking social support, instead of passive strategies, such as avoidance, should reduce anxiety.
(c) Personality traits should be linked to anxiety level, such as neuroticism in a deleterious way.

## 2. Materials and methods

### 2.1. Study design

The present analysis was part of the STERACOVID cohort, an ongoing longitudinal study including psychiatric patients who benefited from clinical teleconsultations by nurses or psychologists during the first lockdown and will continue to until the end of the health crisis. The full study design was recently published (11). The participants were included between January and April 2021. Retrospective data (first lockdown) have been collected in the patients’ medical files. Prospective data was the subject of a standardized collection by telephone, and entered into a database.

In this paper, we used cross-sectional data from a prospective follow-up conducted between 20th January and 5th May 2021, during the COVID-19 crisis. The study was approved by Saint Etienne University Ethics Committee. This trial was registered at clinicaltrials.gov (Identifier: NCT04760795).

### 2.2. Study sample

The research was proposed to all patients who met the inclusion criteria and seen in follow-up consultations by a psychiatrist at the University Hospital of Saint Etienne or at the psychiatric hospital of Lyon. The participants had to be 65 or older and not have a major cognitive disorder that compromised their ability to respond to the scales. They all presented with mental and behavioral disorders, as according to the International Classification of Diseases, Tenth revision edition (ICD-10) criteria. Eight percent of the patients had schizophrenia, schizotypal, or delusional disorder. Fifty-six percent had an affective disorder, including 27% with bipolar disorder. Twenty-six percent had a neurotic, stress-related or somatoform disorder, including 87% of other anxiety disorders. Four percent presented with a personality disorder. Finally, 6% presented with an unspecified mental disorder. These criteria were checked by the patient’s referring psychiatrist.

### 2.3. Data collection

For patients meeting the inclusion criteria and willing to participate in the study, the clinical research associate scheduled a telephone meeting and provided the information note. Participants had a 7-day reflection period.

The data were collected by two psychologists recruited for the study. Interviews were conducted *via* telephone to respect the restrictions linked to the health context.

The telephone interview was split into three parts. All three parts were done in one call. The call was approximately 45 min to 1 hour in length. The telephone appointment had been fixed in advance with the patient by a clinical research associate. The first part collected socio-demographic data, lockdown conditions, and patients’ mood with closed questions, and ordinal scales. The second part consisted of validated scales to assess the state of mental health at the time of the assessment and utilized coping strategies. The third part consisted of scales known to be stable over time, including personality.

### 2.4. Dependent variables

General anxiety was the main outcome measure. The General Anxiety Disorder-7 scale (GAD-7) (12) consists of seven items and measures the presence and severity of anxiety symptoms in the last 7 days, specifically linked to the Diagnostic and Statistical Manual or Mental Disorders 4th version (DSM-IV) criteria. Each of the seven items are scored from 0 to 3. The total score ranges from 0 to 21. Based on this scale participants were divided into two groups: anxious related to GA (ARGA) vs. non-anxious related GA (N-ARGA) with the recommended cut-off of 10 for the identification of GA cases (12).

Anxiety about COVID-19 pandemic (AAC) and subjective mental health (SMH) were also investigated.

Anxiety about COVID-19 pandemic was assessed using a 10-point ordinal scale (from 0, total absence of stress to 10, maximum imaginable stress) answering the question: “on a scale from 0 (total absence of stress) to 10 (maximum imaginable stress) what is your stress level related to the COVID-19 context? SMH was assessed using a 10-point ordinal scale (from 0, the worst possible to 10, the best possible) answering the question: “How do you rate your state of mental health?”

### 2.5. Explanatory variables

Age in years, sex, educational level, and marital status (single/married/widowed/divorced) were collected. Low (below bachelor’s degree) and high (bachelor’s degree and more) educational levels were defined by duration of schooling, with a threshold of 12 years (≥12 years vs. <12 years).

The Brief Coping Orientation to Problems Experienced Inventory (BRIEF-COPE) assesses coping strategies. This scale developed by Caver is an abridged version of the COPE Inventory (13) and has been validated on a French population (14). It includes 14 scales that assess each of the distinct dimensions of coping strategies: (1) active coping, (2) planning, (3) seeking instrumental social support, (4) seeking social emotional support, (5) expressing feelings, (6) behavioral disengagement, (7) distraction, (8) blame, (9) positive reinterpretation, (10) humor, (11) denial, (12) acceptance, (13) religion, and (14) use of substances. Each of these scales includes two items (28 items in total). Depending on the instructions, it can be used in two formats, situational (contextual) or dispositional. In this study, we used the situational form, with the pandemic situation as the reference context. Baumstark et al. (15) suggested grouping the 14 subscales into four factors: social support [includes (3) (4) (13) (5)], problem solving [includes (1) (2)], avoidance [(6) (7) (8) (11) (14)], and positive thinking [(9) (10) (12)]. We used this classification in our analysis.

The Big Five Inventory (BFI) (16), was used to assess personality. This scale was validated in French and consists of 45 items. Each item contains a sentence describing an aspect of personality related to one of the five major dimensions: extraversion, openness, agreeability, consciousness, and neuroticism. An item can reflect either the positive pole or the negative pole of the dimension. The patient is asked to answer on a 5-point Likert scale if he/she disagrees (1) or approves (5) the proposal. The BFI provides a score for each of the five personality dimensions.

Living conditions included: social isolation defined as living alone (yes/no); feeling of loneliness, for which patients were asked if they felt alone [(1) no, (2) sometimes, (3) often, (4) very often]; if they maintain physical contact with proxies (yes/no); the duration of watching TV news [(1) 0 h, (2) <1 h, (3) 1–3 h, and (4) >3 h].

### 2.6. Statistical analyses

Anxious related to general anxiety and N-ARGA were compared using Welch *T* tests for continuous variables for unequal groups, χ^2^ tests for categorical variables, and Wilcoxon rank-sum tests for ordinal variables. We deemed a *p*-value less than 0.05 statistically significant. Associations between GA continuous score and coping strategies on one hand, and personality traits on the other hand were investigated using univariate and multivariate linear regression models using *ImerTest* R package version 3.1-3. Non-linearity, dependence of errors and multicollinearity were systematically checked. A two-step analysis was performed. Firstly, associations of GA with each coping strategy, personality traits, and psychosocial variables were firstly performed using individual linear regression models adjusted for age, sex, and educational level. Secondly, all the associated factors with a conservative *p*-value of 0.05 were introduced into a full multivariate regression model to finally identify the factors independently associated with GA (*p* < 0.05). The same approach was adopted to investigate associations with AAC and psychic health.

The analyses were performed using the R software [version 4.0.3 (2020-10-10); “Bunny–Wunnies Freak out” Copyright^©^ 2020, The R Foundation for Statistical Computing].

## 3. Results

### 3.1. Participants’ characteristics

The study sample included 117 participants among which 68 ARGA and 49 N-ARGA. Characteristics of the participants are displayed in Table 1. There were no significant differences between the two groups on socio-demographic characteristics, psychiatric diagnosis, and psychosocial domain excepted for ARGA participants who consume a significantly higher amount of TV news. (17.9% vs. 2.0% for >3 h, with *p* = 0.043). Compared to N-ARGA, ARGA participants were more likely to experience worse mental health outcomes: worse SMH (5.34, SD 1.56 vs. 6.87, SD 1.64, *p* < 0.001) and a higher of AAC (6.26, SD 1.91 vs. 4.37, SD 1.80, *p* < 0.001). Regarding coping strategies, ARGA participants had a higher mean score for avoidance (18.16, SD 4.01 vs. 14.69, SD 4.19, *p* < 0.001, Cohen’s *d* = 0.38), and a weaker mean score for positive thinking (11.48, SD 3.11 vs. 13.92, SD 3.76, *p* < 0.001, Cohen’s *d* = 0.33). There were no differences for social support and problem-solving strategies. Similarly, ARGA participants experienced a weaker mean score in extraversion (2.83, SD 0.77 vs. 3.17, SD 0.82, *p* = 0.025) and a higher mean score in neuroticism (3.85, SD 0.48 vs. 2.94, SD 0.80, *p* < 0.0001).

**TABLE 1:** Characteristics of the study sample according to general anxiety (GA) status.

	All (*n* = 117)	N-ARGA (*N* = 49)	ARGA (*N* = 68)	*P*-value
**Sociodemographics**				
Age, mean (SD)	77.14 (7.08)	77.63 (6.56)	76.78 (7.47)	0.514
Sex				0.760
Female	83 (70.94%)	36 (73.47%)	47 (69.12%)	
Male	34 (29.06%)	13 (26.53%)	21 (30.88%)	
Educational level				0.868
Low	79 (67.52%)	34 (69.39%)	45 (66.18%)	
High	38 (32.48%)	15 (30.61%)	23 (33.82%)	
Marital status				0.972
Single	18 (15.38%)	7 (14.29%)	11 (16.18%)	
In couple	47 (40.17%)	19 (38.78%)	28 (41.18%)	
Widowed	32 (27.35%)	14 (28.57%)	18 (26.47%)	
Divorced	20 (17.09%)	9 (18.37%)	11 (16.18%)	
**Living conditions**				
Living alone/social isolation				0.675
No	54 (46.15%)	21 (42.86%)	33 (48.53%)	
Yes	63 (53.85%)	28 (57.14%)	35 (51.47%)	
Feeling of loneliness				0.381
None	40 (34.19%)	20 (40.82%)	20 (29.41%)	
Sometimes	41 (35.04%)	18 (36.73%)	23 (33.82%)	
Often	19 (16.24%)	6 (12.24%)	13 (19.12%)	
Very often	17 (14.53%)	5 (10.20%)	12 (17.65%)	
Maintaining physical contact				0.152
No	33 (28.45%)	10 (20.41%)	23 (34.33%)	
Yes	83 (71.55%)	39 (79.59%)	44 (65.67%)	
Duration of watching TV news				**0**.**043**
None	13 (11.21%)	6 (12.24%)	7 (10.45%)	
<1 h	53 (45.69%)	27 (55.10%)	26 (38.81%)	
1–3 h	37 (31.90%)	15 (30.61%)	22 (32.84%)	
>3 h	13 (11.21%)	1 (2.04%)	12 (17.91%)	
**Mental health**				
Subjective mental health score, Mean (SD)	5.98 (1.76)	6.87 (1.64)	5.34 (1.56)	**<0**.**001**
AAC, Mean (SD)	5.47 (2.08)	4.37 (1.80)	6.26 (1.91)	**<0**.**001**
**BRIEF COPE strategies scores**				
Social support, Mean (SD)	16.97 (5.92)	16.78 (5.41)	17.12 (6.29)	0.799
Problem solving, Mean (SD)	8.71 (2.75)	8.47 (2.48)	8.88 (2.94)	0.545
Avoidance, Mean (SD)	16.70 (4.42)	14.69 (4.19)	18.16 (4.01)	**<0**.**001**
Positive thinking, Mean (SD)	12.51 (3.59)	13.92 (3.76)	11.48 (3.11)	**<0**.**001**
**BFI personality traits scores**				
Extraversion, Mean (SD)	2.98 (0.81)	3.17 (0.82)	2.83 (0.77)	**0**.**025**
Agreeableness, Mean (SD)	4.18 (0.46)	4.20 (0.44)	4.17 (0.48)	0.989
Conscientiousness, Mean (SD)	3.70 (0.67)	3.67 (0.76)	3.72 (0.60)	0.350
Neuroticism, (SD)	3.46 (0.78)	2.94 (0.80)	3.85 (0.48)	**<0**.**001**
Openness, (SD)	3.42 (1.07)	3.59 (1.40)	3.30 (0.73)	0.363
**Psychiatric diagnosis**				0.408
Schizophrenia	9 (7.69%)	5 (10.20%)	4 (5.88%)	
Affective disorders	66 (56.41%)	24 (48.98%)	42 (61.76%)	
Neurotic disorders	30 (25.64%)	15 (30.61%)	15 (22.06%)	
Personality disorders	5 (4.27%)	1 (2.04%)	4 (5.88%)	
Mental disorder, unspecified	7 (5.98%)	4 (8.16%)	3 (4.41%)	

The STERACOVID study, 2020, France, *N* = 117. N-ARGA, non-anxious related to general anxiety; ARGA, anxious related to general anxiety; AAC, anxiety about COVID-19 pandemic; BRIEF-COPE, Brief Coping Orientation to Problems Experienced Inventory; BFI, Big Five Inventory; SD standard deviation. Characters in bold are used for significant results *p* < 0.05.

The BRIEF COPE, BFI, and GAD7 scales demonstrated good reliability with respective Cronbach’s alphas of 0.81, 0.79, and 0.94.

### 3.2. Factors associated with general anxiety during COVID-19 crisis

Results of linear regression models for GA are presented in Table 2. For individual models (model 1), avoidance coping strategy was associated with an increase in GAD-7 score (β = 0.73, *p* < 0.0001), positive thought strategy with a decreased GAD-7 score (β = −0.73, *p* < 0.0001), while social support and problem-solving strategies were not associated with GA. Regarding personality traits, extraversion was associated with a lower GAD-7 score (β = −1.55, *p* = 0.0483) and neuroticism with a higher GAD-7 score (β = 5.65, *p* < 0.0001). Most of the psychosocial variables were associated with GAD-7 score: a deep feeling of loneliness (β = 3.92, *p* = 0.0472), and a long-time exposure to TV news (β = 6.88, *p* = 0.0072) were associated with a higher score in GAD-7. Conversely, maintaining physical contact was associated with a lower score in GAD-7 (β = −3.15, *p* = 0.0227).

**TABLE 2:** Parameter estimates of the linear regression models for general anxiety (GA).

	Model 1^a^				Model 2^b^				Model 3^c^			
	β	SD	*t* value	*P*-value	β	SD	*t* value	*P*-value	β	SD	*t* value	*P*-value
BRIEF COPE social support	0.04	0.11	0.42	0.6723								
BRIEF COPE problem solving	0.20	0.24	0.83	0.4076								
BRIEF COPE avoidance	0.73	0.12	5.75	**<0**.**0001**	1.73	0.50	3.45	**0**.**0008**	1.72	0.52	3.31	**0**.**0013**
BRIEF COPE positive thinking	-0.73	0.16	-4.59	**<0**.**0001**	-0.66	0.54	-1.21	0.2247	-0.66	0.58	-1.15	0.2535
BFI extraversion	-1.55	0.77	-1.99	**0**.**0483**	0.94	0.63	1.49	0.1393	0.87	0.66	1.31	0.1931
BFI agreeableness	-1.05	1.38	-0.76	0.4475								
BFI conscientiousness	0.55	0.95	0.58	0.5603								
BFI neuroticism	5.65	0.60	9.33	**<0**.**0001**	4.64	0.65	7.04	**<0**.**0001**	4.56	0.68	6.67	**<0**.**0001**
BFI openness to experience	-0.48	0.59	-0.82	0.4131								
**Feeling of loneliness**												
Sometimes	2.75	1.46	1.87	*0*.*0635*	0.40	1.09	0.36	0.7127	0.36	1.11	0.33	0.7439
Often	3.32	1.84	1.80	0.0739	0.73	1.35	0.54	0.5894	0.77	1.37	0.56	0.5753
Very often	3.92	1.95	2.00	**0**.**0472**	-0.08	1.44	-0.06	0.9515	-0.10	1.51	-0.07	0.9478
Living alone	-1.10	1.25	-0.88	0.3803								
**Duration of watching TV news**												
<1 h	0.90	1.98	0.45	0.6487	0.77	1.45	0.53	0.5957	0.46	1.54	0.30	0.7673
1–3 h	3.04	2.08	1.46	0.1469	1.99	1.52	1.31	0.1941	1.87	1.60	1.17	0.2461
>3 h	6.88	2.51	2.73	**0**.**0072**	3.94	1.95	2.01	**0**.**0463**	3.66	2.01	1.83	0.0709
Maintain physical contact	-3.15	1.36	-2.31	**0**.**0227**	-0.36	1.06	-0.34	0.7310	-0.49	1.10	-0.44	0.6612
Age					-0.07	0.06	-1.13	0.2598	-0.07	0.06	-1.04	0.3004
Sex					1.35	1.05	1.27	0.2040	1.35	1.09	1.24	0.2176
Educational level					0.83	0.99	0.83	0.4035	0.83	1.01	0.83	0.4107
Schizophrenia									0.47	1.87	0.25	0.8024
Affective disorders									0.88	2.05	0.43	0.6676
Neurotic disorders									2.98	3.02	0.99	0.3252
Personality disorders									0.18	2.69	0.07	0.9456
Mental disorder, unspecified									0.47	1.87	0.25	0.8024

The STERACOVID study, 2020, France (N = 117). BRIEF-COPE, Brief Coping Orientation to Problems Experienced Inventory; BFI, Big Five Inventory; SD, standard deviation.

^a^Model 1: individual models: individual linear regression models adjusted for age, sex, and educational level.

^b^Model 2: full model: full multivariate linear regression model including BRIEF COPE avoidance, BRIEF COPE positive thinking, BFI extraversion, BFI neuroticism, feelings of loneliness, duration of watching TV news, and if they maintain physical contact, adjusted for age, sex and, educational level.

^c^Model 3: Model 2 and adjusted for psychiatric diagnosis.

Characters in italics are used for results near to significance 0.05 < *p* < 0.1; characters in bold are used for significant results *p* < 0.05.

The results of the full model (model 2) showed that avoidance coping strategy, a high level of neuroticism, and long-time spent watching TV news remained independently associated with a higher level of GA (β = 1.73, *p* = 0.0008, β = 4.64, *p* < 0.0001, and β = 3.94, *p* = 0.0463, respectively).

The results of the full model adjusted for psychiatric diagnosis (model 3) showed that avoidance coping strategy and high neuroticism remained independently associated with higher GA but not associated with news watching, for which we found only a trend toward significance (β = 1.72, *p* = 0.0013, β = 4.56, *p* < 0.0001, and β = 3.66, *p* = 0.0709, respectively).

### 3.3. Factors associated with anxiety about COVID-19 pandemic and with subjective mental health

The results of the full model for AAC without adjustment for psychiatric diagnosis (model 2) showed that avoidance coping strategy and neuroticism were independently associated with higher AAC (β = 0.44, *p* = 0.0278 and β = 0.52, *p* = 0.0386, respectively), whereas living alone was associated with lower AAC (β = −0.73, *p* = 0.0387). With adjustment for psychiatric diagnosis (model 3), the association between neuroticism and higher AAC did not reach but was close to significance (β = 0.47, *p* = 0.0705) (Appendix Table A). For SMH, the model 2 showed that positive thinking coping strategy was associated with better SMH (β = 0.43, *p* = 0.0101), whereas neuroticism and a deep feeling of loneliness were associated with poorer SMH (β = −0.62, *p* = 0.0018, and β = −1.05, *p* = 0.0163, respectively). The same associations were observed in model 3, with adjustment for psychiatric diagnosis (Appendix Table B).

## 4. Discussion

The aim of our study was to identify the factors associated with higher GA, during the COVID-19 pandemic, in older patients followed-up in psychiatric centers.

As expected, we found that coping strategies, personality, and living conditions were associated with GA during the pandemic period. Specifically, higher GA was associated with less positive thinking coping, more avoidance, a lower level of extraversion, a higher level of neuroticism, more time watching the news, a higher feeling of loneliness, and a lack of physical contact.

### 4.1. Factors associated with general anxiety during COVID-19 crisis

#### 4.1.1. General anxiety and coping

Previous studies had already linked anxiety with less effective coping strategies during the COVID-19 crisis (9, 10, 17).

Similar to our study, these studies did not show an association between active coping, such as problem solving or seeking social support on anxiety (10, 17), whereas these strategies are typically considered to be functional. Coping depends on various variables specific to the context and to the individual himself. The uncontrollable and unprecedented aspects of the pandemic did not allow the use of previous resolution schemes. This could explain why we did not find an association with problem-solving strategies. In addition, lockdowns and protective measures prevented the establishment of social support.

Also consistent with previous findings, our study showed an association between more frequent use of avoidance strategy and a higher level of anxiety. Indeed, Orhan et al. (10) showed in older adults with mental disorders–exclusively bipolar disorders–that passive coping style, including avoidance, was associated with more GA during the pandemic. In the study conducted by Mariani et al. (17), participants were younger and without psychiatric disorders. They found a positive correlation between anxiety and avoidance, but also between anxiety and emotion-oriented coping. However, they used another coping scale, Coping Inventory for Stressful Situations (CISS) (18) with different dimensions of coping strategies than us to consider coping. Indeed, our scale did not allow the independent study of emotion-oriented coping. This difference could also explain why our findings regarding the link between positive thinking and lower levels of anxiety had not been shown in previous work.

Another reason that could explain this difference is that previous studies were conducted during the first lockdown, opposite to our work that was done later in the pandemic. Coping may have varied between the two lockdowns because of the announcement of vaccines and more hope for the future.

#### 4.1.2. General anxiety and personality

As in the study conducted by Orhan et al. (10), we found more GA in people with a higher level of neuroticism. In addition, we found that extraversion was linked to GA during the pandemic. In the general population outside a pandemic situation, Jylhä and Isometsä (19) also showed strong links between higher neuroticism and anxiety and, to a lesser extent, lower extraversion. In both their work and our study, extraversion was no longer significantly associated with anxiety in the final model, possibly due to the negative correlation between neuroticism and extroversion scores.

Nevertheless, higher neuroticism and more frequent use of avoidance strategy remained significantly associated with higher GA in the final model despite the close links between coping and personality (20). Therefore, neuroticism and avoidance each appeared to play an independent role in GA during the pandemic.

#### 4.1.3. General anxiety and living conditions

The other factors associated with higher GA during the pandemic were related to living conditions. These factors are important because they are able to be adjusted in case of a new lockdown.

The first association found among these factors was exposure to TV news. Limiting the time watching news to reduce anxiety had already been reported in previous work carried out during the first lockdown in the general population (21). Our study seems to confirm this finding for older patients with psychiatric disorders. The effect is strong since the relationship is still present in the multivariate model, and a trend toward significance persists after adjustment for the psychiatric diagnosis. Indeed, situations that are difficult to understand can be experienced as unreal and cause fascination and astonishment, leading patients to remain transfixed in front of the news. In this context, offering prevention and psychoeducation can be a real lever for reducing anxiety.

We failed to show an association between social isolation and GA level. In contrast, a greater feeling of loneliness was associated with higher GA. This could mean that loneliness rather than living alone during the pandemic-could be associated with greater GA. On the other hand, the lack of physical contact also seemed to be linked to greater GA. Another work showed an increase in feelings of loneliness and anxiety among older people during the first lockdown compared to before the pandemic. However, they did not study the association between anxiety and loneliness (22).

### 4.2. Other measures

Additional analyzes on AAC and SMH complemented these results. Compared to GA, we obtained different results on the association between AAC and coping or social isolation. Socially isolated patients appeared to have less AAC. In addition, patients who used more frequently the social support strategy were more anxious. Taken together and paradoxically, these results suggested that in a pandemic context, the most isolated patients who relied less on proxies could also be those who suffered less from lockdowns. We can assume that patients living alone are less afraid of transmitting the virus to their relatives or of being infected by them. In addition, it seems possible that patients who live alone or who rely less on others have less changed their lifestyle during lockdowns and therefore are less anxious about the context.

Additionally, using more frequently the problem-solving strategy was also associated with greater AAC. As proposed before, the situation being unprecedented, it seemed difficult to set up problem solving strategies in an uncertain future.

These results are contrary to those obtained by Budmir et al. (23) in an adult population without psychiatric disorders. However, they noted that the active coping strategy was the one that had the weakest effect on the various measures of mental health and also hypothesized that the situation pandemic was not very controllable by individuals. Finally, the search for social support in younger adults without psychiatric disorder was undoubtedly favored by a good use of new technologies less well mastered by our older patients.

Conversely, the problem-solving strategy was associated with better SMH. Likewise, a trend toward significance was found for the association between better perceived mental health and use of social support coping strategy. We could therefore assume that these strategies seem globally effective in protecting mental health, but not in reducing anxiety in a pandemic context. This hypothesis would be a line of work, in particular for future longitudinal study.

### 4.3. Strengths and limitations

Several limitations need to be highlighted to ensure the correct interpretation of our findings. The latest results from secondary analysis were based on unvalidated ten-point ordinal scales. Moreover, the feeling of loneliness was not measured with a validated scale. In contrast, we used robust and validated scales for our primary measures such as coping, personality, and GA.

Another limitation was heterogenous psychiatric diagnoses in our population. Furthermore, some patients had an unspecified mental disorder. However, most of them had either mood or anxiety disorders, so this population represented the reality of clinical practice. The specificity of our population was the originality of the study: indeed, few previous studies had focused on psychiatric older patients specifically. But it is also a limitation because we cannot generalize our results to the entire adult psychiatric population or the general population.

Finally, we did not make a longitudinal comparison with measurements before the pandemic because this crisis couldn’t have been anticipated. Therefore, we cannot conclude about a causal link in the associations found. We will continue to monitor the patients, allowing us to have interesting longitudinal data between during pandemic and after pandemic.

Our study has, however, several strengths. To our knowledge, this is the first to focus on the mental health of older adults later in the pandemic. In addition, assessing the patients by phone reduced the recruitment bias, compared to online surveys. Moreover, the telephone interviews collected by trained psychologists, allowed a more precise assessment than when the patient completed the questionnaires alone.

### 4.4. Conclusion and clinical implications

In conclusion, our study identified factors associated with a poor experience of lockdowns and pandemic crisis in older patients followed-up in a psychiatric center.

Some factors can already help in identifying at-risk patients, while other ones can guide support in the event of a new pandemic context. In a similar situation, special attention should be paid to patients with a high level of neuroticism and a high feeling of loneliness. Support could aim at helping patients use more functional strategies. For example, encouraging patients to reduce avoidance strategies in favor of positive thinking and reducing time spent watching the news, could be interesting prevention perspectives.

Longitudinal data are being collected and will allow us to know the long-term effects of the COVID-19 outbreak on these patients.

## Data availability statement

The raw data supporting the conclusions of this article will be made available by the authors, without undue reservation.

## Ethics statement

The studies involving human participants were reviewed and approved by the Ethics Committee of the University Hospital of Saint-Étienne. This committee covers the ethical approval for the three sites of our data collection. All procedures follow the Declaration of Helsinki and the International Conference on Harmonization (ICH) Good Clinical Practice Guidelines. The patients have received an informed written information notice. The STERACOVID study is registered in the Clinical Trials database (Current Controlled Trials NCT04760795 http://clinicaltrials.gov/show/NCT04760795). The Ethics Committee waived the requirement of written informed consent for participation because the research uses only usual clinical practice questionnaires.

## Author contributions

IR conceived the idea for the study, helped to draft the manuscript, and managed the design and coordination of the study. EP participated in its design, coordination management, and drafted the manuscript for submission to Frontiers in Psychiatry. AE performed the statistical analyzes and helped to draft the manuscript. RB helped to draft the manuscript. J-MD, AB, MH, BL, CB, LS, CP, HM, and CL-G participated in its design and coordination. IR, AB, HE, CP, and CL-G are responsible for the participants’ inclusion at the University Hospital of Saint-Étienne. J-MD and MH are responsible for the participants’ inclusion at the Vinatier Hospital. All authors approved the final version of the manuscript.

## Appendix

**APPENDIX TABLE A:** Parameter estimates of the linear regression models for Anxiety about COVID-19 (AAC) pandemic.

Anxiety about COVID-19	Model 1^a^				Model 2^b^				Model 3^c^			
	β	SD	*t* value	*P*-value	β	SD	*t* value	*P*-value	β	SD	*t* value	*P*-value
BRIEF COPE social support	0.08	0.03	2.64	**0.0280**	0.30	0.19	1.68	0.0950	0.30	0.20	1.51	0.1353
BRIEF COPE problem solving	0.15	0.07	2.10	**0.0094**	3.53	0.21	1.63	0.1044	0.34	0.22	1.55	0.1235
BRIEF COPE avoidance	0.18	0.04	4.40	**<0.0001**	0.44	0.19	2.23	**0.0278**	0.46	0.20	2.27	**0.0256**
BRIEF COPE positive thinking	−0.13	0.05	−2.60	**0.0106**	−0.33	0.21	−1.59	0.1148	−0.33	0.22	−1.48	0.1419
BFI extraversion	−0.27	0.24	−1.13	0.2605								
BFI agreeableness	−0.49	0.42	−1.15	0.2491								
BFI conscientiousness	0.14	0.29	0.49	0.6249								
BFI neuroticism	0.87	0.23	3.69	**0.0003**	0.52	0.25	2.09	**0.0386**	0.47	0.26	1.83	0.0705
BFI openness to experience	−0.08	0.18	−0.46	0.6461								
**Feeling of loneliness**												
Sometimes	0.25	0.45	0.56	0.5732								
Often	−0.62	0.57	−1.08	0.2794								
Very often	0.89	0.60	1.65	0.1465								
Living alone	−1.08	0.37	−2.87	**0.0048**	−0.73	0.35	−2.09	**0.0387**	−0.81	0.37	−2.20	**0.0298**
**Time in watching TV news**												
<1 h	−0.33	0.62	−0.53	0.5909								
1–3 h	0.68	0.65	1.04	0.2980								
>3 h	0.93	0.78	1.18	0.2376								
Maintain physical contact	−0.71	0.42	−1.65	0.1015								
Age					0.00	0.02	−0.01	0.9985	0.00	0.02	0.06	0.9518
Sex					−0.08	0.41	−0.02	0.9840	−0.07	0.43	−0.16	0.8765
Educational level					0.39	0.37	1.05	0.2926	0.41	0.38	1.08	0.2834
Schizophrenia									0.44	0.65	0.68	0.5015
Affective disorders									0.57	0.71	0.80	0.4269
Neurotic disorders									1.51	1.02	1.48	0.1425
Personality disorders									0.44	0.65	0.68	0.5015
Mental disorder, unspecified									0.57	0.71	0.80	0.4269

The STERACOVID study, 2020, France (*N* = 117). BRIEF-COPE, Brief Coping Orientation to Problems Experienced Inventory; BFI, Big Five Inventory; SD, standard deviation.

^a^Model 1: individual models: individual linear regression models adjusted for age, sex, and educational level.

^b^Model 2: full model: full multivariate linear regression model including BRIEF COPE problem-solving, BRIEF COPE avoidance, BRIEF COPE positive thinking, BFI neuroticism, and social isolation, adjusted for age, sex, and educational level.

^c^Model 3: model 2 and adjusted for psychiatric diagnosis.

Characters in italics are used for results near to significance 0.05 < *p* < 0.1; characters in bold are used for significant results *p* < 0.05.

**APPENDIX TABLE B:** Parameter estimates of the linear regression models for psychic health.

Psychic health	Model 1^a^				Model 2^b^					Model 3^c^			
	β	SD	*t* value	*P*-value	β	SD	*t* value	*P*-value		β	SD	*t* value	*P*-value
BRIEF COPE social support	0.05	0.02	1.96	*0.0517*									
BRIEF COPE problem-solving	0.20	0.06	3.24	**0.0015**	0.25	0.16	1.55	0.1227		0.27	0.16	1.64	0.1045
BRIEF COPE avoidance	−0.06	0.03	−1.58	0.1167									
BRIEF COPE positive thinking	0.24	0.04	5.95	**<0.0001**	0.43	0.16	2.62	**0.0101**		0.50	0.17	2.87	**0.0051**
BFI extraversion	0.75	0.19	3.85	**0.0001**	0.02	0.19	0.13	0.8949		0.11	0.20	0.53	0.5951
BFI agreeableness	0.55	0.36	1.52	0.1310									
BFI conscientiousness	0.74	0.24	3.05	**0.0028**	0.30	0.21	1.41	*0.1611*		0.26	0.22	1.21	0.2307
BFI neuroticism	−1.07	0.18	−5.68	**<0.0001**	−0.62	0.19	−3.20	**0.0018**		−0.54	0.20	−2.73	**0.0076**
BFI openness to experience	0.29	0.15	1.87	*0.0628*									
**Feeling of loneliness**													
Sometimes	−0.21	0.37	−0.51	0.5667	0.31	0.32	0.97	0.3299		0.28	0.32	0.85	0.3953
Often	−0.98	0.37	−2.06	**0.0412**	−0.46	0.40	−1.16	0.2485		−0.48	0.40	−1.21	0.2303
Very often	−1.75	0.49	−3.52	**0.0006**	−1.05	0.43	−2.44	**0.0163**		−0.93	0.44	−2.10	**0.0381**
Social isolation	−0.32	0.33	−0.98	0.3267									
**Duration of watching TV news**													
<1 h	−0.27	0.53	−0.50	0.6148	−0.09	0.43	−0.20	0.8357	0.17		0.46	0.37	0.7160
1–3 h	−0.96	0.56	−1.71	0.0890	−0.70	0.45	−1.54	0.1259	−0.47		0.47	−1.00	0.3189
>3 h	−1.47	0.68	−2.16	**0.0326**	−1.01	0.57	−1.80	0.0745	−0.92		0.57	−1.61	0.1112
Maintain physical contact	0.59	0.36	1.61	0.1103									
Age					0.00	0.02	−0.04	0.9684	0.00		0.02	0.03	0.9784
Sex					−0.35	0.32	−1.08	0.2792	−0.29		0.33	−0.89	0.3776
Educational level					−0.41	0.29	−1.43	0.1551	−0.40		0.29	−1.37	0.1745
Schizophrenia									−0.44		0.51	−0.86	0.3924
Affective disorders									−0.91		0.56	−1.62	0.1078
Neurotic disorders									−1.11		0.85	−1.30	0.1966
Personality disorders									−1.09		0.76	−1.44	0.1525
Mental disorder, unspecified									−0.44		0.51	−0.86	0.3924

The STERACOVID study, 2020, France (*N* = 117). BRIEF-COPE, Brief Coping Orientation to Problems Experienced Inventory; BFI, Big Five Inventory; SD, standard deviation.

^a^Model 1: individual models: individual linear regression models adjusted for age, sex, and educational level.

^b^Model 2: full model: full multivariate linear regression model including BRIEF COPE problem-solving, BRIEF COPE avoidance, BRIEF COPE positive thinking, BFI neuroticism, and social isolation, adjusted for age, sex, and educational level.

^c^Model 3: Model 2 and adjusted for psychiatric diagnosis.

Characters in italics are used for results near to significance 0.05 < *p* < 0.1; characters in bold are used for significant results *p* < 0.05.
